# Circular RNA screening identifies circMYLK4 as a regulator of fast/slow myofibers in porcine skeletal muscles

**DOI:** 10.1007/s00438-021-01835-5

**Published:** 2021-11-16

**Authors:** Haigang Cao, Jieming Liu, Tianning Du, Yihao Liu, Xiaoyu Zhang, Yuan Guo, Jie Wang, Xiaomin Zhou, Xiao Li, Gongshe Yang, Xin’e Shi

**Affiliations:** 1 Key Laboratory of Animal Genetics, Breeding and Reproduction of Shaanxi Province, College of Animal Science and Technology, Northwest A&F University, No. 22 Xinong Road, Yangling, Xianyang, 712100 Shaanxi China; 2 Laboratory of Animal Fat Deposition and Muscle Development, College of Animal Science and Technology, Northwest A&F University, No. 22 Xinong Road, Yangling, Xianyang, 712100 Shaanxi China

**Keywords:** High throughput sequencing, CircMYLK4, Pig, Meat quality, Muscle fiber type

## Abstract

The type of myofiber is related to the quality of meat. The slow oxidized myofiber helps to increase the tenderness and juiciness of muscle. Numerous studies have shown that circRNA plays a key role in skeletal muscle development. However, the role of circRNA in porcine skeletal myofiber types is unclear. In this study, we performed high-throughput RNA sequencing to study the differential expression of circRNA in the longissimus dorsi and the soleus muscle. A total of 40,757 circRNAs were identified, of which 181 were significantly different. Interestingly, some circRNAs were involved in metabolism pathways, AMPK, FoxO, and PI3K-Akt signaling pathways. Besides, we focused on a novel circRNA-circMYLK4. By injecting circMYLK4-AAV into piglets, we found that circMYLK4 significantly increased the mRNA and protein levels of the slow muscle marker genes. In summary, our study laid an essential foundation for further research of circRNA in myofiber type conversion and higher meat quality.

**Supplementary Information:**

The online version contains supplementary material available at 10.1007/s00438-021-01835-5.

## Introduction

Meat quality has been a major focus over the last decades because of both increasing living standards and population growth. As one of the dietary structures in our daily life, the quality of pork affects everyone’s health. Therefore, improving quality without decreasing meat production is a promising research direction for porcine breeding scientists. Pork quality is influenced by multiple factors, and a growing number of studies have shown that muscle fiber types are closely related to quality (Lefaucheur 2010). Compared to fast muscle fiber, slow muscle fiber improves meat quality by affecting meat pH (Ryu and Kim 2006; Choi et al. 2007; Kim et al. 2013b), meat color (Renerre 1990; Kauffman et al. 1998; Kim et al. 2010), and water-holding capacity (Larzul et al. 1997; Kim et al. 2013b). Hence, increasing the proportion of slow muscle fibers is a powerful strategy to improve meat quality.

It is generally believed that the total number of muscle fibers remains basically unchanged after the birth of an animal but that the muscle fiber type transform during the growth process. Transformation is the result of multiple factors, such as internal regulatory factors, which are mainly associated with signaling pathways and related cytokines, among which circular RNAs (circRNAs) play an important role. CircRNAs are a type of covalently closed circular RNA molecules formed by reverse splicing of the precursor mRNA without 5′ cap and 3′ poly (A) tail structures (Kristensen et al. 2019). Previous studies have shown that circRNAs are present in tissues and cells of various biological organisms. For example, circRNAs are abundantly expressed in skeletal muscles of monkey and directly or indirectly regulate important factors of muscle (Abdelmohsen et al. 2015). Moreover, circLMO7 regulates the proliferation and differentiation of myoblasts by sponging miR-378a-3p (Wei et al. 2017). Additionally, the protein translated by circZNF609 is involved in the growth and development of muscle (Legnini et al. 2017). More importantly, other studies have shown that circRNAs mainly regulate skeletal muscle growth development and muscle fiber type conversion during 0–30 days after birth and regulate skeletal muscle glucose metabolism and calcium ion signal during 30–240 days, but their regulatory mechanism is still unclear (Liang et al. 2017). These results suggest that circrna plays an important role in skeletal muscle development and muscle fiber type conversion; however, the roles and regulatory mechanisms of many circRNAs remain unknown.

In this study, a high-throughput sequencing technique was used to construct a cDNA library of skeletal muscle, including fast muscle (longissimus dorsi, LD) and slow muscle (soleus, Sol). After quality control filtration of the data, 181 differentially expressed circRNAs were identified by screening. Interestingly, we were attracted to a special circRNA, circMYLK4, which was highly expressed in slow muscle. Thus, we investigated the effects of circMYLK4 on muscle fiber types in pigs by injecting adeno-associated virus (AAV). As a result, circMYLK4 promoted the development of slow muscle fibers. These results provide a useful resource for further exploration of circRNA in transformation of muscle fiber types and improvement of meat quality.

## Materials and methods

### Ethics statement

All animal surgeries were performed in compliance with the ARRIVE guidelines (Kilkenny et al. 2010). All animals care, slaughter and sample collection procedures were carried out in strict accordance with the protocol approved by the Institutional Animal Care and Institutional Ethic Committee of Northwest A&F University (NWAFU-314020038).

### RNA extraction and RNase R treatment

The longissimus dorsi and soleus muscle samples were taken from three 180-day-old healthy male large white pigs of the same parity and body weight. There pigs were castrated under anesthesia (surgery was performed under sodium pentobarbital anesthesia, and all efforts were made to minimize suffering), and they were housed under standard environmental conditions (including unregulated room temperatures and natural light) and were fed a standard diet three times a day and watered add libitum. Total RNA was extracted from muscle samples by the TRIzol reagent according to the manufacturer’s instructions (Takara, Kyoto, Japan). The quality and concentration of RNA were detected by a NanoDrop 2000 instrument (Thermo Scientific, Waltham, MA, USA). Subsequently, ribosomal RNA (rRNA) was removed by Epicenter Ribo-zero rRNA Removal Kit (Epicenter, USA). Then, the residue RNAs were treated with RNase R (Epicenter, USA) to degrade the linear RNAs and were purified using RNeasy MinElute Cleanup Kit (Qiagen, Germany).

### Library construction and sequencing

Ribosome RNA and linear RNA were removed to retain circRNAs. We used fragmentation buffer (Ambion, Foster City, CA, USA) to fragment the enriched circRNA and reverse transcribed the circRNA into cDNA using random primers. The second strand of cDNA was synthesized by DNA polymerase I, RNase H, dNTPs and buffer. The double-stranded cDNA was purified with the QiaQuick PCR extraction kit (Qiagen, USA), end repaired, polyadenylated, and ligated to Illumina sequencing adapters. Then, we used Uracil-*N*-Glycosylase (UNG) to digest the second-strand cDNA. The fragments were purified by VAHTSTM DNA Clean Beads and enriched by PCR amplification. Finally, the library products were sequenced via Illumina HiSeqTM2500.

### Quality control, sequence mapping and circRNA prediction

The original image file (BCL) obtained by sequencing was base-recognized and converted into raw data of FASTQ format. Then, clean data was obtained by filtering raw data, de-joining sequences, treating low-quality reads, quality assessing, and removing ribosome RNA. The clean data were compared with the reference genome twice. First, a normal alignment was performed by Tophat2 software (Kim et al. 2013a), and the fusion gene was not included for comparison so that the reads that could not be directly compared to the genome were obtained. Then the Tophat-fusion module of Tophat2 software was used to perform fusion gene alignment on unmapped reads, to obtain potential circular reads. The Tophat-fusion results were filtered according to circRNA reads with reverse splicing points and sequences of splicing sites that are usually GT/AG, to identify circRNA more accurately (Kim and Salzberg 2011; Kim et al. 2013a; Zhang et al. 2014).

### Expression profiling and analysis of differentially expressed circRNAs

We used HTseq software to calculate the count value for each circRNA, and then further calculated its RPM value, and also counted the number of splicing site sequences. DESeq was used in subsequent analysis to calculate the *p*-value and *q*-value of the gene in the comparison group, and the differentially expressed circRNAs between the samples were selected by the difference multiplier (log2 (Fold change) > 1) and the significance level (*q*-value < 0.05) (Wang et al. 2010).

### Network construction and KEGG enrichment analysis

We used miRanda, RNAhybrid, and TargetScan software to predict miRNA binding to exonic circRNA (John et al. 2004; Bernhart et al. 2006; Wang et al. 2019a), and then we used Cytoscape software to construct a circRNA-miRNA interaction network. Additionally, miRanda, pita, and RNAhybrid software was used to predict the target genes of these miRNAs, and then biological pathway enrichment analysis was conducted on the target gene sets based on the Kyoto Encyclopedia of Genes and Genomes (KEGG) biological pathway database (Ogata et al. 1999).

### In vivo injection of circMYLK4-AAV

Construction of the circMYLK4 overexpression vector and virus packaging were completed by Guangzhou geneseed. The circMYLK4-AAV virus titer packaged by geneseed is 1 × 10^13^ GC/mL. We selected five 12-day-old male DLY (Duroc × Landrace × Yorkshire) piglets for gastrocnemius injection. Each piglet was injected with 50 μL of virus titer of 1 × 10^13^ GC/mL in the left leg (diluted 50 μL of virus into 2 mL for easy injection), and the same amount of control virus was injected in the right leg. The piglets were sacrificed four weeks after injection, and the gastrocnemius, semitendinosus, semimembranosus and soleus muscles were separately collected for subsequent experiments.

### Real-time quantitative polymerase chain reaction (RT-qPCR) and Western blot

RT-qPCR was performed on an Applied Biosystems real-time quantitative PCR machine using SYBR green master mix (Vazyme, China) according to the manufacturer’s protocol. All primers used in RT-qPCR are shown in Table 1. Protein extraction and Western blot analysis of tissues were performed according to our previous methods (Wang et al. 2019b). Antibodies against MyHCI were from Developmental Studies Hybridoma Bank (DSHB, USA). Antibodies against β-tubulin were from Boster.

**Table 1:** Primer sequences for RT-qPCR

Genes	Primer sequences
*CircBRWD1*	F: AAAGGAGCATCAAAGCAACCA
	R: TCGGAAGATCACTATCATCTGGAT
*CircCSRP3*	F: CGTCTACCACGCAGAAGAAATC
	R: ACTCTCTGATCAAGCCGGCAC
*CircMYLK4*	F: ACCAGCTCCATTCCAGGCTC
	R: CTCCGAGGATCTCCGTTCTG
*CircIGFN1a*	F: AGATCTGCCTGAAGTACGGC R: GGGATGTCGTCCACCAGC
*CircIGFN1b*	F: GTGCCCGACTTTGAGCAGAA R: CTGTGCAGGGACTGCTTACC
*CircIGFN1c*	F: GAGGAATGGGGTCTGGCTTT R: CAGACCCAGCCTTTCCTGT
*CircIGFN1d*	F: GCGCGGAGAGGACAGTGA R: CCTCTTCTCCCAGAAAGCCCT
*CircIGFN1e*	F: CCAGGTTCGCTAGATCCCAA R: ACTAGTGACGCCTAGGGGAG
*CircIGFN1f*	F: TTCTTCCCCAACCATCCTGTC R: CCAACCTTCAACCAGGTGCT
*Tnni1*	F: CCACAGTCTGCAGTCCACA
	R: CAGGTAGCGAGCCTTCTCAG
*Tnnc1*	F: ATGGTTCGGTGCATGAAGGA
	R: ATCCTCTGTGATGGTCTCGC
*Tnnt1*	F: GCAGAGAGAGCTGAGCAACA
	R: TCCTCTCCCATGTGGTCGAT
*Myl3*	F: AGGACTTTGTGGAAGGGCTG
	R: TCTTGCCCAGCCATCAACTT
*PGC-1α*	F: ACGAGCGTCATTCAGGAGC
	R: AGCACACTCGATGTCAGTCC
*Tfam*	F: GACTACTGCGTCTGCACCTT
	R: AGCAACTCTTCAGACCTCGC
*Atp5o*	F: TTGCTTGCTGAAAACGGTCG
	R: GAGATGCAGTGGTGACGGAA
*Cycs*	F: TGGTTAAACTTGAATGCGGAGTG
	R: GGCTCATGCCTTAACAGGCT
*Ndufa5*	F: ACTGTGAGTAGTGCTCCCCT
	R: GAGCGAGGCTGTCTAGGTTC
*MyHCI*	F: AAGGGCTTGAACGAGGAGTAGA
	R: TTATTCTGCTTCCTCCAAAGGG
*β-actin*	F: GGACTTCGAGCAGGAGATGG
	R: AGGAAGGAGGGCTGGAAGAG

### Statistical analysis

The experimental data are expressed as the mean ± SEM. All experimental data were analyzed by ANOVA and significance test using GraphPad Prism 7. *P < 0.05, **P < 0.01.

## Results

### Overview of circRNA profiles in LD and Sol skeletal muscle

**Fig. 1:**
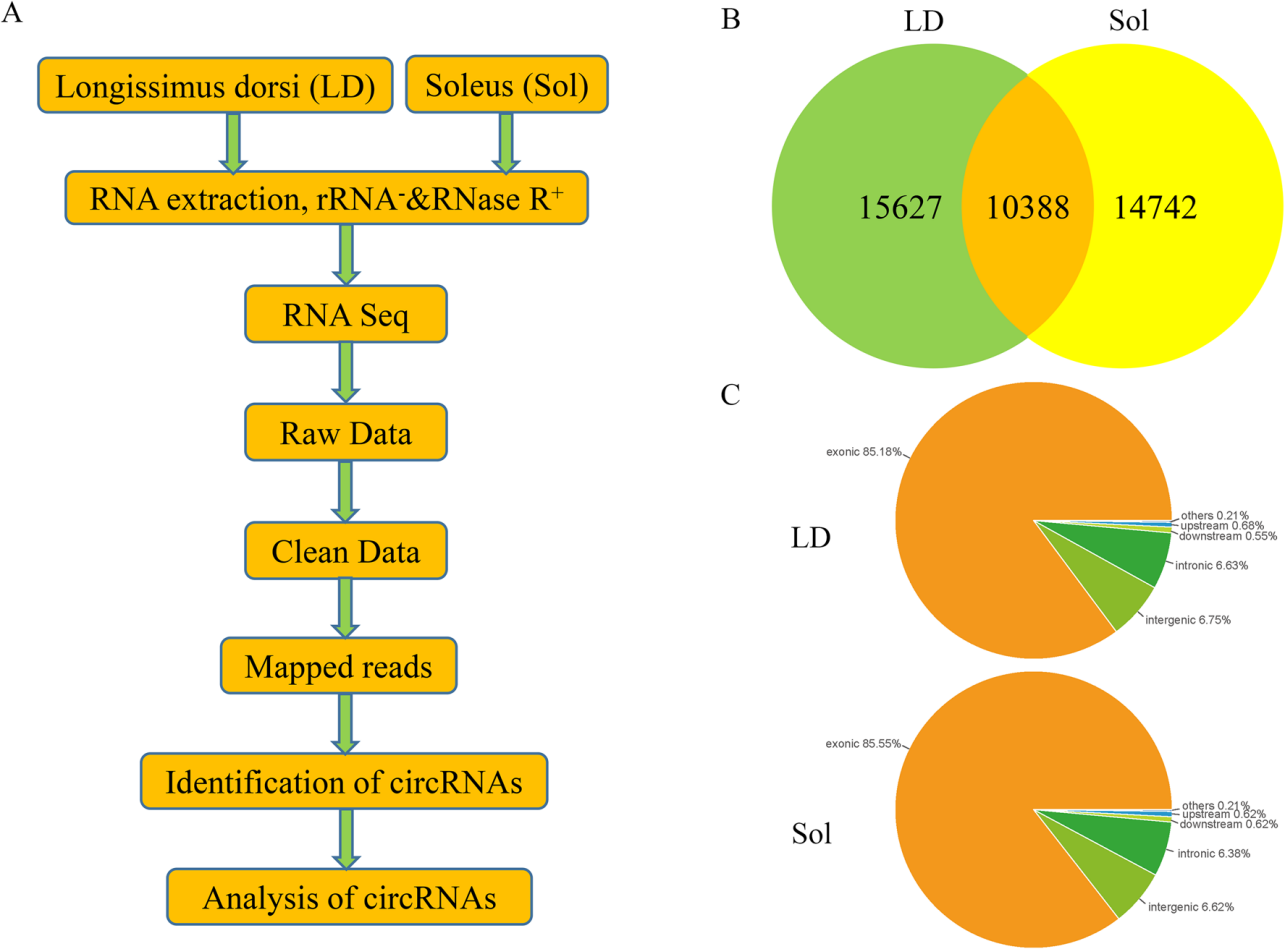
Identification of circular RNAs in LD and Sol skeletal muscles. **A** Pipeline for circRNA identification. **B** Venn diagram showing the overlap of annotated circRNAs among LD and Sol skeletal muscles. **C** Origin of circRNAs described in this study in the *Sus scrofa* genome

To determine the identity, abundance and differential expression of circRNAs in different types of skeletal fibers, we profiled circRNAs in slow-type-enriched Sol and fast-type-enriched LD of large white pigs. Ribo-depleted total RNA-Seq libraries were prepared and sequenced according to the flow chart (Fig. 1A). We identified a considerable number of RNAs in LD and Sol muscles when considering total RNA libraries (Table 2). A total of 40,757 candidate circRNAs were identified by at least one unique back-spliced read. We found that 15,627 and 14,742 circRNAs were specifically expressed in LD and Sol muscles respectively, and 10,388 were expressed in both muscles (Fig. 1B). For both the LD and Sol samples, the circRNAs are mostly comprised of exonic, intronic and intergenic sequences, as determined based on their mapping to the genome, whereas a smaller fraction of circRNAs also contains upstream, downstream and UTRs (Fig. 1C).

**Table 2:** Summary of reads mapping to the reference genome and identification of circular RNAs

Samples	LD	Sol
Raw reads	99,987,084	82,541,576
Clean reads	86,645,908	72,841,678
Mapped reads	73,928,442 (85.52%)	63,016,935 (86.77%)
Mapped reads (unfusion)	62,626,818 (72.45%)	53,855,038 (74.15%)
Candidate back-spliced junction reads	1,000,194 (1.154%)	880,632 (1.209%)
Realign post reads	424,828 (0.490%)	328,353 (0.451%)
Circular RNA number	26,015	25,130
Circular RNA number (reads > 1)	20,351	19,483

### Identification and characteristics of circRNAs expressed in LD and Sol

**Fig. 2:**
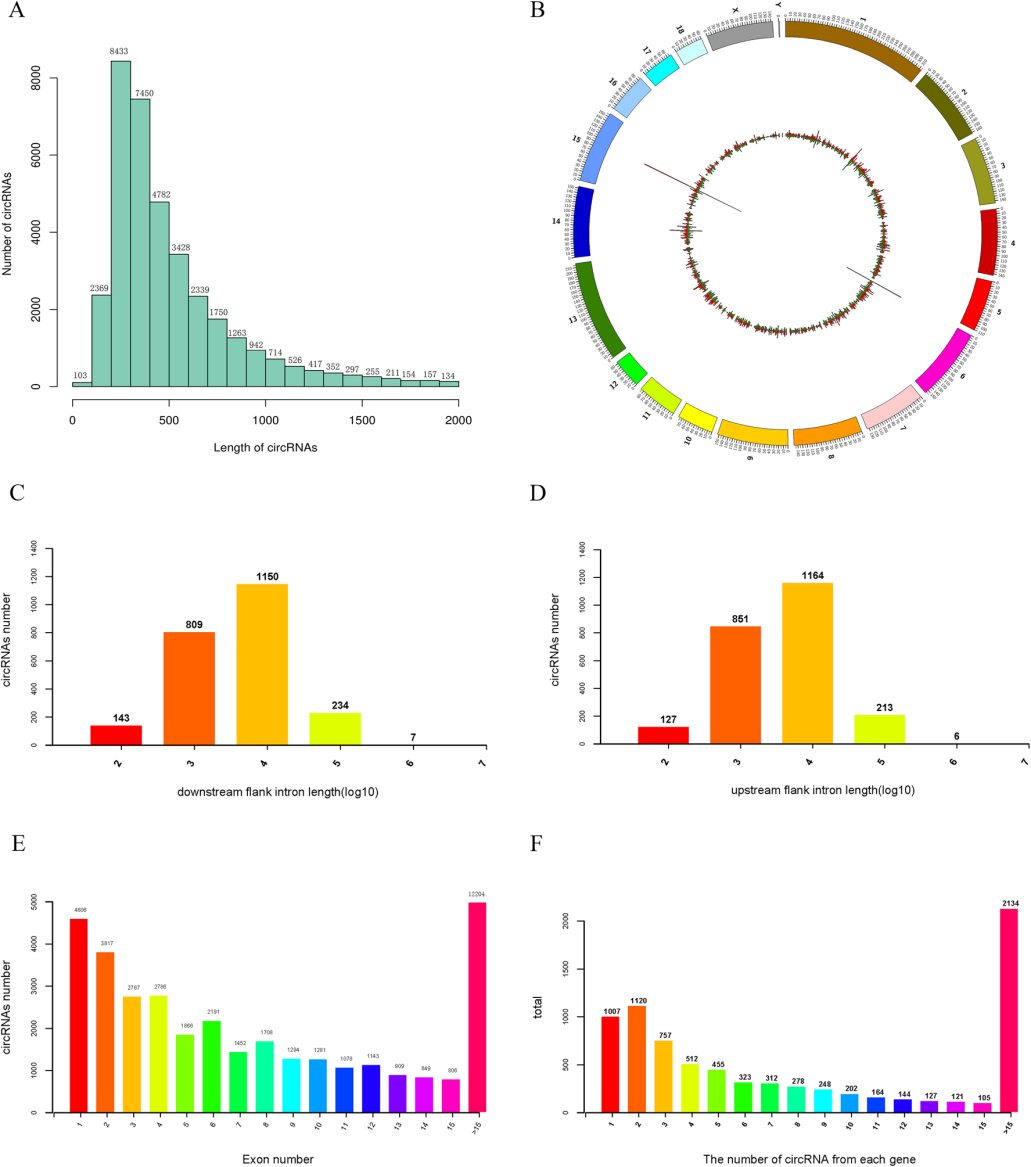
General characteristics of circular RNAs in pigs. **A** Length distribution of circRNAs. **B** Circos plot showing the distribution of circRNAs on different chromosomes. **C, D** Length of circRNAs flanking introns. **E** circRNAs that contained varying numbers of exons. **F** Distribution of the number of circRNAs per gene

To further estimate the characteristics of circRNAs, we analyzed the circRNA sequences. Standard metrics to characterize the length of circRNAs detected in this study (minimum, maximum, mean, median, and total length) are provided in Table 3. Most circRNAs were no more than 1000 nucleotides (nt), and the median length was 439 nt (Fig. 2A). According to their host gene location, the 40,757 circRNAs were widely distributed across chromosomes except for the Y chromosome, and the number of circRNAs increased with chromosome length (Fig. 2B). According to the histogram depicting flanking intron lengths, the length of flanking intron regions of most circRNAs was approximately 10^3^–10^4^ nt, indicating that long flanking introns are necessary for circRNA formation (Fig. 2C, D). A total of 21,193 (52.0%) circRNAs were composed of one to eight exons, among which 4606 (11.3%) contained one exon. Furthermore, 12,204 (29.9%) circRNAs contained more than fifteen exons (Fig. 2E). We identified 40,757 circRNAs generated from 8009 host genes, indicating that most genes can produce multiple circRNAs. There were 1007 (12.6%) circRNA-producing genes that generated a single circRNA, whereas 26.6% produced more than fifteen (Fig. 2F).

**Table 3:** Results from the assembly of circRNAs

Item	circRNA	Min. length	Mean length	Median length	Max. length	Total length
Number	40,757	34	2734	439	99,934	111,433,971

### Analysis of differentially expressed circRNA between LD and Sol

**Fig. 3:**
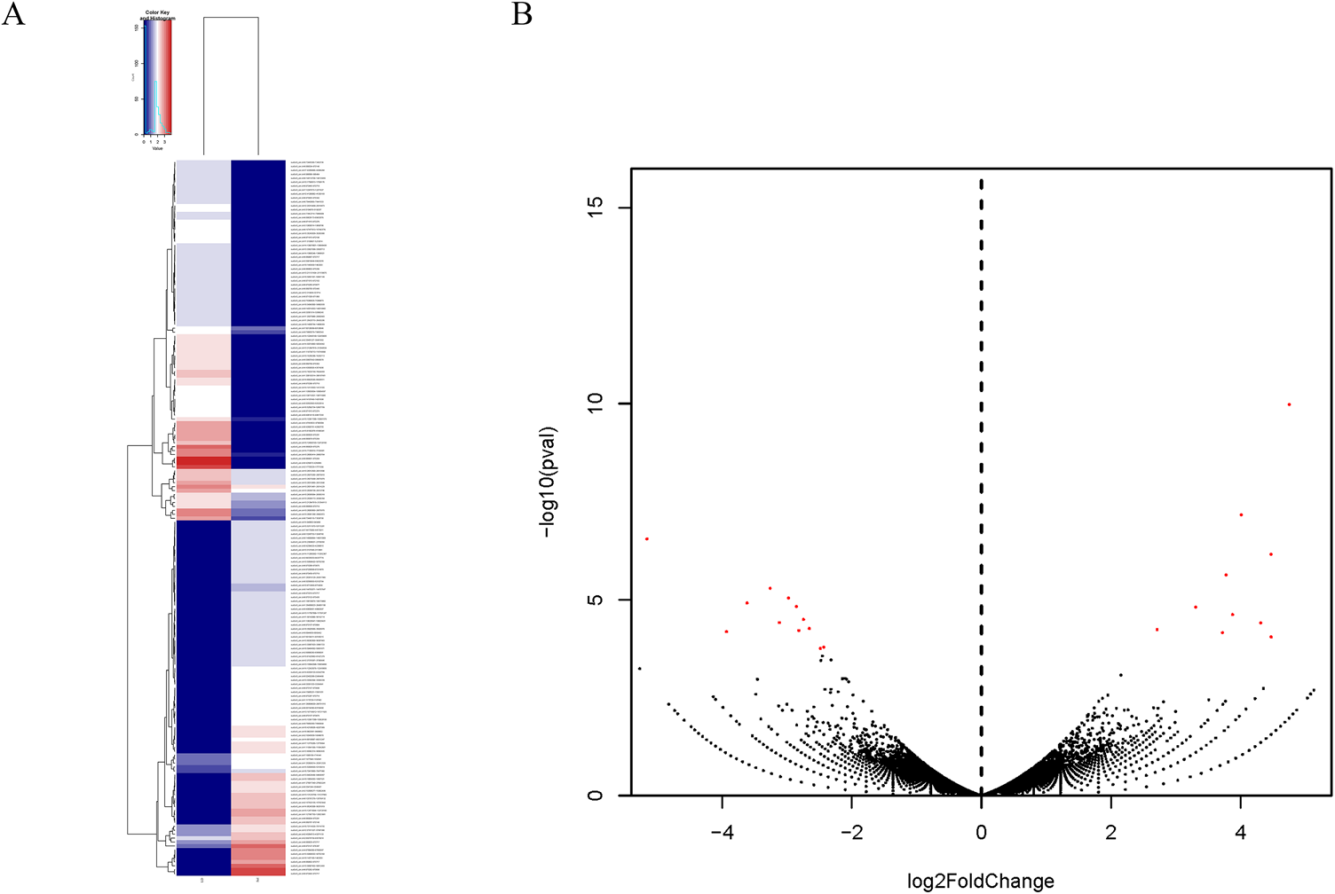
Differential circular RNA expression between LD and Sol skeletal muscles. **A** Cluster heat-map of differentially expressed circRNAs from each sample. **B** Volcano plots showing –log_10_ (pval) versus log_2_ fold difference in circRNAs abundance in RPM between LD and Sol skeletal muscles. Red dots denote significantly differently expressed circRNAs

Based on the analysis of circRNA expression, we found 181 significantly differentially expressed circRNAs when comparing the libraries derived from the LD and Sol (Additional File). To further explore the potential functions of circRNA, we constructed a clustered heatmap (Fig. 3A). Although several circRNAs showed the same expression level, most of them were differentially expressed between the LD and Sol. Based on the expression levels of circRNAs in paired samples (LD to Sol ratio), 90 circRNAs were downregulated, and 91 circRNAs were upregulated in the LD (Fig. 3B).

### Construction of circRNA-miRNA interaction network

**Fig. 4:**
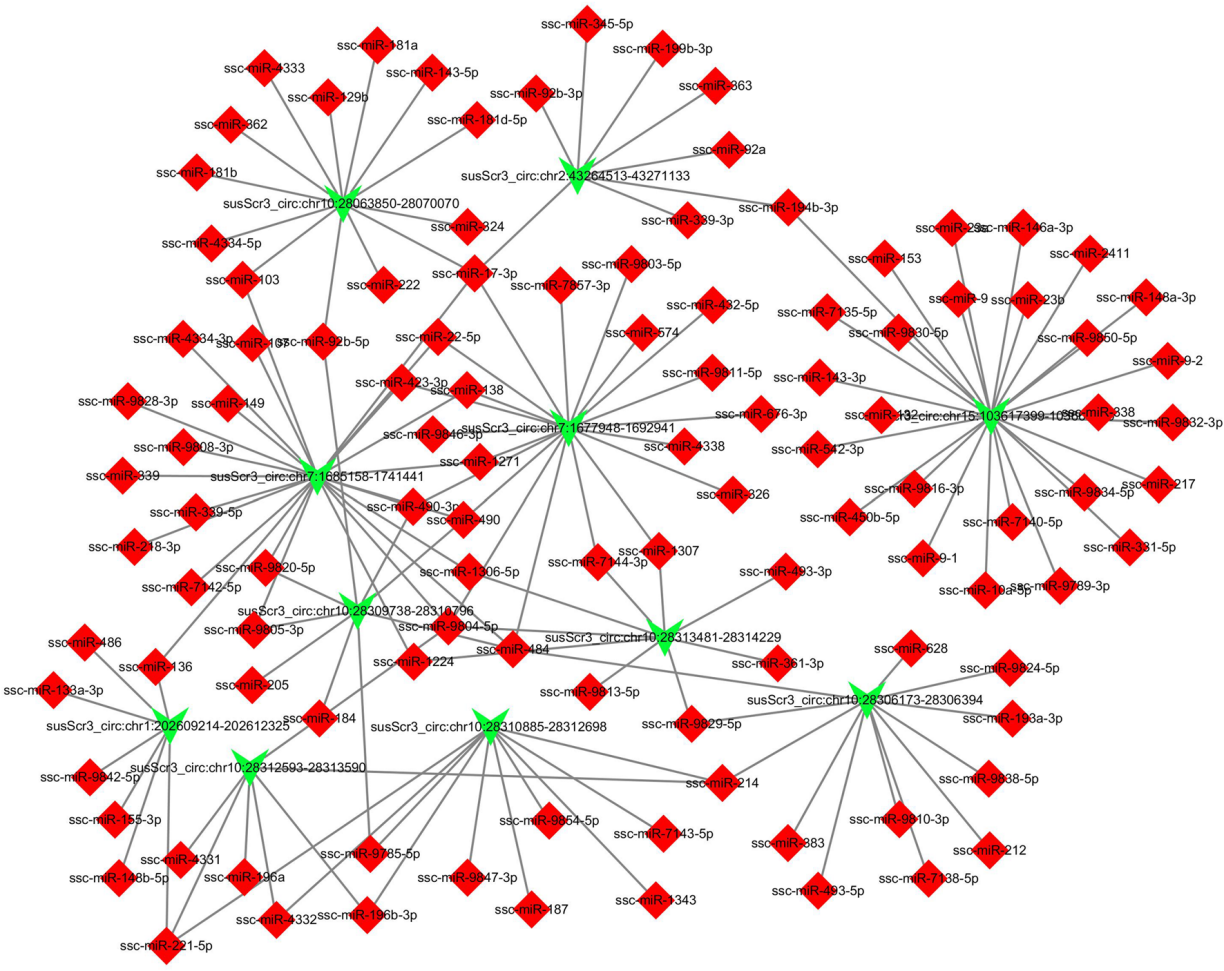
Co-expression network of the miRNAs and candidate circRNAs. Red squares represent miRNAs. Green arrows represent circRNAs

A previous study has shown that circRNAs composed of only exons can play a role in the cytoplasm by sponging microRNA (miRNA) (Wang et al. 2021). We randomly selected 11 differentially expressed exonic circRNAs and predicted the miRNAs they might sponge by miRanda, RNAhybrid and TargetScan. Then, we constructed an interactive network between these 11 circRNAs and the sponged miRNAs (Fig. 4). The whole interaction network included 11 circRNAs, 111 miRNAs and 146 relationship lines.

### KEGG pathway enrichment analysis

**Fig. 5:**
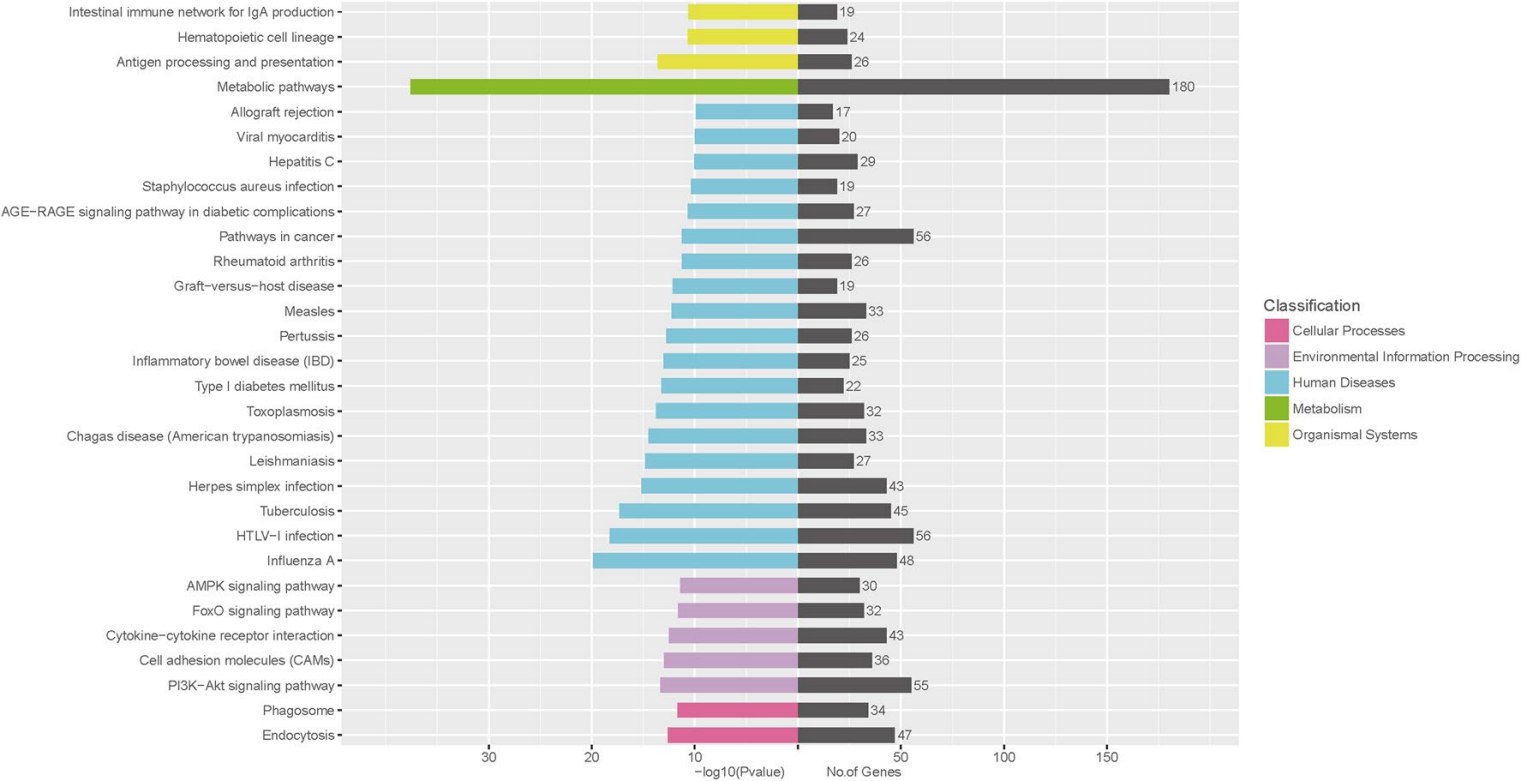
Kyoto Encyclopedia of Genes and Genomes pathways. KEGG pathway enrichment analysis of target genes of candidate circRNAs

To further analyze the regulatory mechanisms of differentially expressed circRNAs in different types of skeletal muscle fibers, we predicted the sponge miRNAs of the differentially expressed circRNAs and analyzed the target genes of miRNAs. Then, we performed pathway enrichment analysis of the predicted target genes (Fig. 5). A total of 30 signaling pathways were enriched for these genes, among which metabolic pathways were the most abundant pathway. Markedly, some pathways were closely related to muscle fiber development, resulting in the transformation of muscle fiber types, such as the AMPK signaling pathway, FoxO signaling pathway, and PI3K-Akt signaling pathway.

### Validation of differentially expressed circRNAs by RT-qPCR

**Fig. 6:**
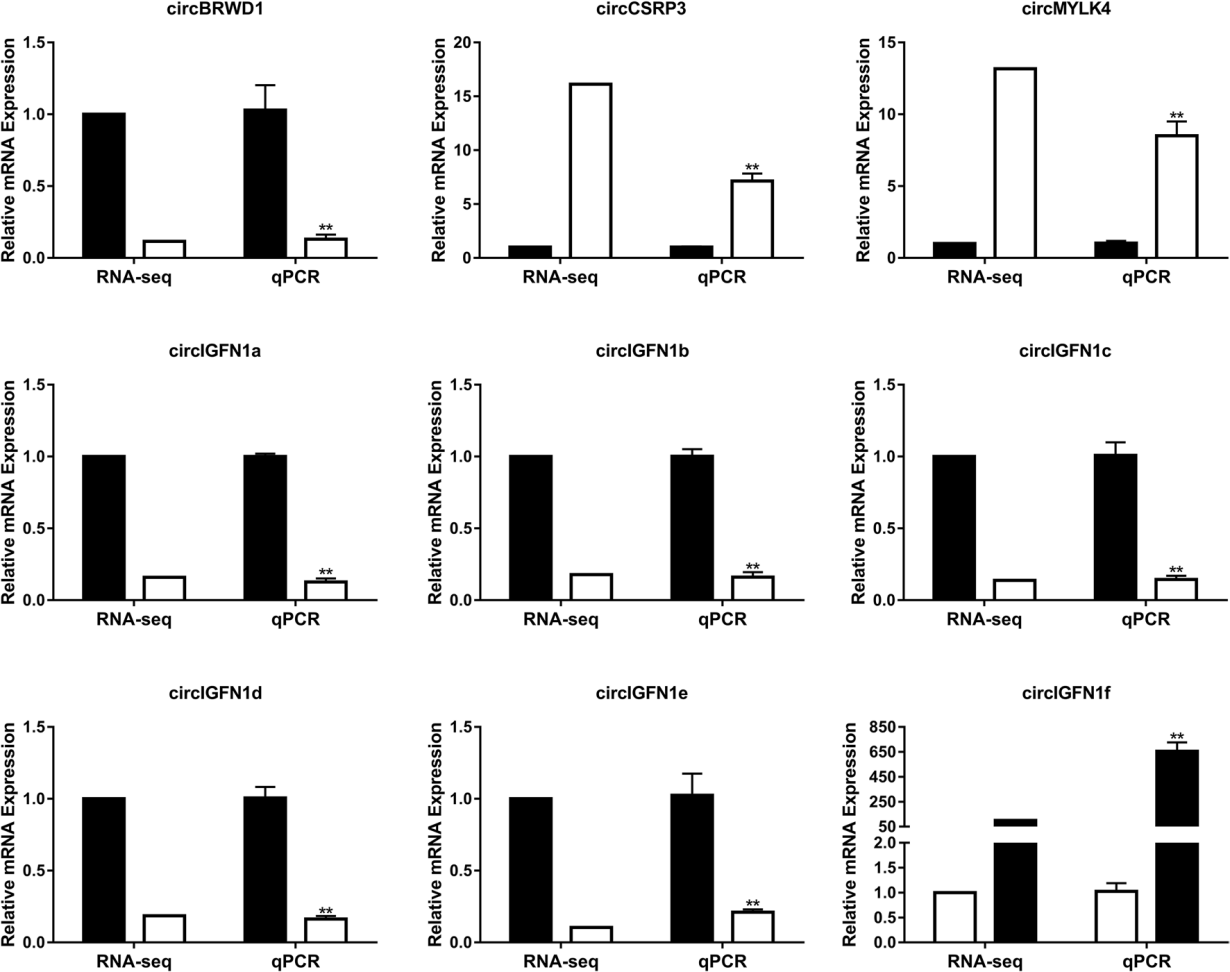
RT-qPCR validation of nine differentially expressed circular RNAs between LD and Sol. Black indicates the tissue of LD, and white indicates the Sol. The data presented in the *Y* axis represent the relative expression of both RPM and RT-qPCR and are expressed as the mean ± SEM. *P < 0.05, **P < 0.01. *N* = 3

To validate the data reliability of differentially expressed circRNAs detected by sequencing, we randomly selected 9 differentially expressed circRNAs and amplified their junction regions using specific RT-qPCR primers. RT-qPCR revealed two upregulated and seven downregulated circRNAs, as shown in Fig. 6, which clearly showed that expression patterns of the nine selected circRNAs were consistent with the results of RNA-Seq. These results demonstrated the accuracy and reliability of high-throughput sequencing.

### Overexpression of circMYLK4 promotes the development of slow muscle fibers

**Fig. 7:**
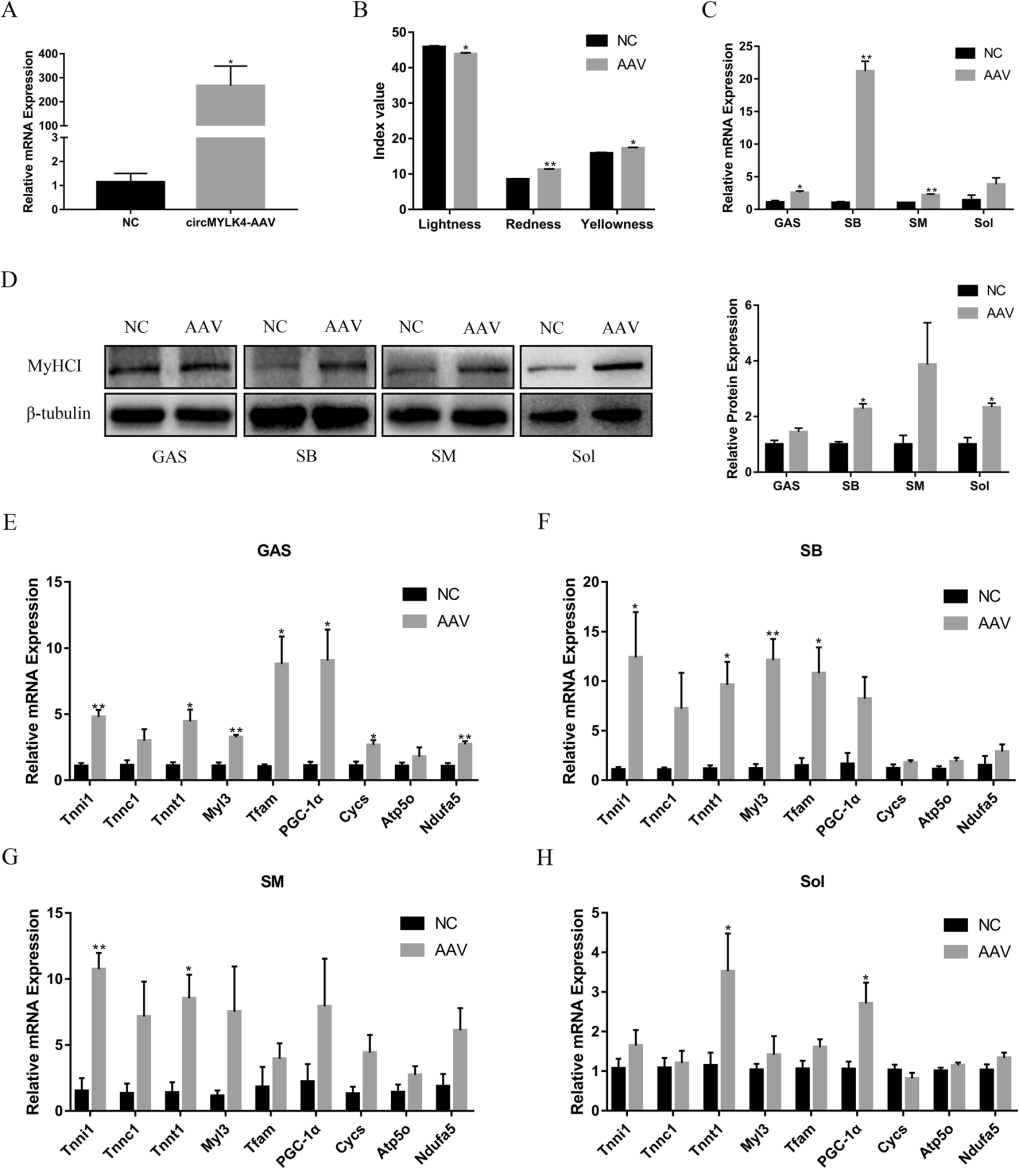
circMYLK4 promotes the formation of slow muscle fibers. **A** Relative expression levels of circMYLK4 in AAV and control groups. **B** Skeletal muscle color test results after overexpression of circMYLK4. **C** The mRNA expression levels of MyHC I in different skeletal muscle tissues after overexpression of circMYLK4. **D** The protein expression and gradation analysis results of MyHC I in different skeletal muscle tissues after overexpression of circMYLK4. **E–H** The mRNA expression of slow fiber-related genes and mitochondria-related genes in different skeletal muscle tissues after overexpression of circMYLK4

We analyzed novel circRNAs in many aspects and selected circMYLK4, which was named after its host gene MYLK4 located on chromosome 7, for its high expression in Sol for further analysis. To investigate whether this circRNA could be a regulatory factor during muscle fiber development, we injected piglets with circMYLK4-AAV. Then, we collected gastrocnemius (GAS), semimembranosus (SB), semitendinosus (SM) and soleus (Sol) samples for analysis 4 weeks after injection. The expression level of circMYLK4 in AAV group was significantly higher than that in control group (Fig. 7A). The results of the meat color assessment showed that circMYLK4 reduced the brightness of meat and improved the redness and yellowness of meat (Fig. 7B). In support of this phenomenon, circMYLK4 overexpression significantly increased expression levels of the slow muscle marker gene MyHC I, which was detectible at both the mRNA and protein levels (Fig. 7C, D). In addition, we also measured the mRNA of fiber type-related genes (Tnnt1, Tnni1, and Tnnc1), mitochondrial biogenesis genes (PGC-1α and Tfam), and mitochondrial respiratory chain genes (Atp5o, Cycs, and Ndufa5) by RT-qPCR. As expected, circMYLK4 overexpression increased the expression of the above genes (Fig. 7E–H). Collectively, these results established that circMYLK4 promoted the development of slow muscle fibers by regulating the expression of mitochondrial-related factors.

## Discussion

The characteristics of skeletal muscle fiber are associated with the quality of meat (Joo et al. 2013). Generally, skeletal muscle fibers can be divided into fast muscle fibers and slow muscle fibers according to their morphological and functional characteristics (Bassel-Duby and Olson 2006). Slow muscle fibers are characterized by high content of mitochondria, lipids and myoglobin and a small diameter, while fast muscle fibers have the opposite characteristics (Holloszy and Coyle 1984). Almost all skeletal muscles are composed of slow muscle fibers and fast muscle fibers. Studies have shown that a higher content of slow muscle fibers makes the meat ruddy, fresh and juicy, which improves the flavor of meat, while a higher content of fast muscle fibers makes the meat white and leads to reduced quality (Joo et al. 2013). Therefore, increasing the ratio of slow muscle fiber can improve meat quality. CircRNAs were originally thought to be a by-product of splicing (Hsu and Coca-Prados 1979), but with the rapid development of high-throughput sequencing technology, their true nature is gradually becoming well known. In recent years, the research on circRNAs has mainly focused on human diseases in medicine, and there have been less studies on skeletal muscle fibers in agricultural animals. Recent studies have found that circRNAs can act as novel regulatory factors in skeletal muscle (Legnini et al. 2017). CircRNAs are abundant in skeletal muscle, conserved between species and regulated in myogenesis and muscular disease. Specifically, several circRNAs have been reported to regulate the proliferation and differentiation of myoblasts (Legnini et al. 2017; Wei et al. 2017), which means that circRNA has a very important significance in muscle growth and development. Therefore, the in-depth exploration of circRNAs involved in muscle fiber development is a new direction for meat quality improvement research.

In this study, a total of 40,757 circRNA were identified from the LD and Sol muscles, of which 10,388 were co-expressed in the two muscles. Further, 181 differentially expressed circRNAs in the LD and Sol were identified. CircRNA may partially participate in the regulation of porcine skeletal muscle fiber types through signaling pathways such as AMPK, FoxO, and PI3K-Akt. The AMPK signaling pathway regulates the differentiation directions of myoblasts and changes the types of myofiber (Chalkiadaki et al. 2014). The FoxO signaling pathway is regulated by a variety of phosphorylated kinases and plays an important role in the proliferation and differentiation of myoblasts and the transformation of myofiber type (Schachter et al. 2012). PI3K-AKT signaling pathway is closely related to the repair and regeneration of skeletal muscle, not only promoting skeletal muscle protein synthesis, but also activating the proliferation of myosatellite cells, which in turn promotes skeletal muscle regeneration and repair of damage (Bai et al. 2015; Fu et al. 2019). More important, a novel circRNA, circMYLK4, could promote the development of slow muscle fibers. Overall, our findings provide scientific evidence that circRNAs regulate muscle fiber types.

In recent years, studies on circRNA in pigs have mainly focused on the sequencing identification stage. Huang studied the circRNA expression profiles of 70-day-old Jinhua pigs and Landrace pigs and identified 84,864 circRNAs, of which 366 were differentially expressed and mainly enriched in lipid metabolism pathways (Huang et al. 2018). This provides basic data for further study of the biological function of porcine liver circRNA. Liang performed a circRNA genome-wide analysis of nine organs (muscle, fat, liver, heart, spleen, lung, kidney, ovary, and testis) and skeletal muscles of three different development stages in Guizhou mini-pigs. A total of 5934 circRNAs were identified, of which 149 were related to muscle development, and the first porcine circRNAs database was constructed (Liang et al. 2017). Sun performed high-throughput sequencing of longissimus miRNA, lncRNA, and circRNA of Landrace and Lantang pigs, and the results showed that 236 differentially expressed circRNAs were screened, among which 40 participated in sponge modulators of 26 miRNA-mediated ceRNA interactions (Sun et al. 2017). Their study revealed a new post-transcriptional regulatory layer that could promote the understanding of the molecular mechanisms of muscle fiber development in different breeds of pigs. Shen studied the circRNA expression profiles of the psoas major muscle and the longissimus dorsi muscle of 178-day-old Qingyu pigs and identified 810 circRNAs, of which 137 were differentially expressed (Shen et al. 2019). These circRNAs may be involved in the regulatory network of muscle fiber type. In our study, a total of 40,757 circRNAs were identified, of which 181 were significantly differentially expressed. Overall, our identification results enriched the pig circRNA database, providing a basic reference for studying pig muscle fiber type transformation and improving meat quality.

In the characterization of circRNAs, we found that the length of circRNAs is mainly distributed approximately 400 bp, and the length of circRNAs is positively correlated with the corresponding chromosome length. This finding is similar to previous observations of bovine skeletal muscle samples (Wei et al. 2017). This finding potentially indicates that the length and number of circRNAs are common features of skeletal muscle tissue. Additionally, we found that the length of the upstream and downstream flanking introns of circRNAs is mostly approximately 10,000 bp. This is caused by the main loop-forming mechanism of circRNA, in which the flanking intron region contains repeats or complementary sequences, such as SINE (Veno et al. 2015) or ALU sequences (Jeck et al. 2013; Liang and Wilusz 2014; Zhang et al. 2014). In addition, the flanking introns need to be long enough to facilitate this event. Our results indicate that one parental gene can produce multiple circRNA isoforms through alternative splicing (Zhang et al. 2014).

Skeletal muscle fiber types are regulated by a number of signaling pathways. Transformation of skeletal muscle fiber types is accompanied by changes in mitochondria and metabolism. Studies have shown that succinate induces skeletal muscle fiber remodeling by promoting mitochondrial biogenesis and aerobic oxidation (Wang et al. 2019c). Additionally, ferulic acid promotes the formation of slow muscle fibers by activating Sirt1 and AMPK to upregulate the expression of PGC-1α (Chen et al. 2019). 6-Gingerol stimulates AMPK/PGC-1α signaling pathway and accelerates a fast-to-slow-fiber type transition and muscle oxidative metabolism by increasing plasma adiponectin and muscle AdipoR1 (Liu et al. 2019). Kitamura found that Foxo1 ablation in skeletal muscle results in increased formation of MyoD-containing (fast-twitch) muscle fibers and altered fiber type distribution at the expense of myogenin-containing (slow-twitch) fibers (Kitamura et al. 2007). In our study, we found that target genes of differentially expressed circRNA-sponge miRNAs were enriched in the AMPK, FoxO and PI3K-Akt signaling pathways, potentially suggesting that these circRNAs regulate muscle fiber types through the indicated signaling pathways. Although there is no clear evidence that PI3K-Akt plays a role in muscle fiber types, some studies have shown that the PI3K-Akt signaling pathway is related to skeletal muscle development and atrophy (Stitt et al. 2004; Yu et al. 2015).

Studies have shown that circMYLK produced by human myosin light chain kinase (MYLK) can competitively bind to miR-29a, abolishing miR-29a inhibition of VEGFA/VEGFR2 and downstream Ras/ERK signaling pathways, thereby promoting epithelial-mesenchymal transition and bladder cancer development (Zhong et al. 2017). In this sequencing analysis, we found that circMYLK4 produced by myosin light chain kinase family member 4 (MYLK4) in pigs was significantly differentially expressed in different types of skeletal muscle fiber in pigs. Interestingly, circMYLK4 has a completely different sequence from circMYLK and is considered a new circRNA. By injecting circMYLK4-AAV into piglets, we found that circMYLK4 promotes the expression of slow muscle fiber marker genes and mitochondria-related genes. Our results require further study of the mechanism by which circMYLK4 upregulates slow muscle fibers. In the future, we will explore the pathways though which circMYLK4 influences muscle fiber types. It is of great theoretical significance to clarify the mechanism of circMYLK4 regulating the transformation of pig skeletal muscle fiber types for improving pork quality.

**Fig. 8:**
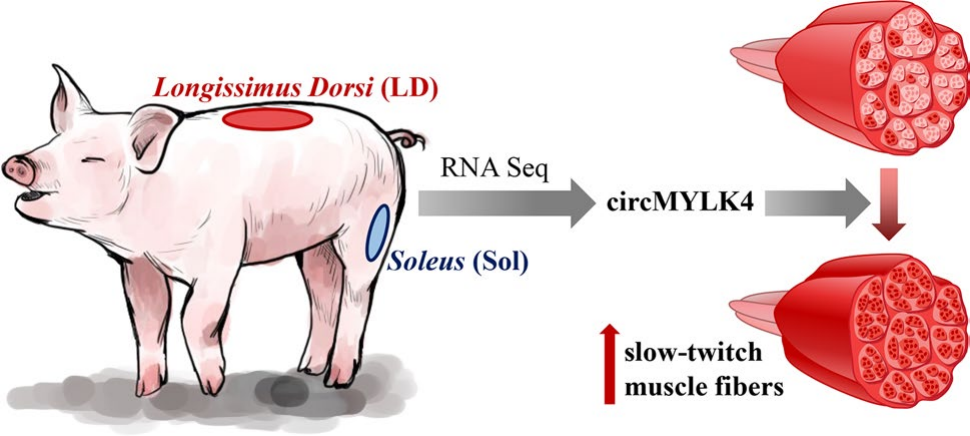
Schematic diagram of circMYLK4 affecting muscle fiber types

In this study, we found 181 differentially expressed circRNAs between the longissimus dorsi and soleus muscles of pigs and identified a novel circRNA, circMYLK4 that promoted the formation of oxidized fibers (Fig. 8). These findings provide a theoretical basis for further study of myofiber type conversion and higher meat quality.

## Supplementary Information

Below is the link to the electronic supplementary material.

Supplementary file1 Supplementary File: Differentially expressed circRNA between LD and Sol. (XLSX 28 KB)

## Abbreviations

CircRNA: Circular RNA
LD: Longissimus dorsi
Sol: Soleus
GAS: Gastrocnemius
SB: Semimembranosus
SM: Semitendinosus
AAV: Adeno-associated virus
AMPK: Adenosine 5′-monophosphate (AMP)-activated protein kinase
FoxO: Forkhead box O
PI3K-Akt: Phosphatidylinositol 3 kinase-protein kinase B
MyHC I: Myosin heavy chain I
Tnnt1: Troponin T1
Tnni1: Troponin I1
Tnnc1: Troponin C1
PGC-1α: Peroxisome proliferator-activated receptor gamma coactivator 1
Tfam: Transcription Factor A Mitochondrial
Atp5o: ATP Synthase Peripheral Stalk Subunit OSCP
Cycs: Cytochrome C Somatic
Ndufa5: NADH-Ubiquinone Oxidoreductase Subunit A5
MYLK4: Myosin light chain kinase family member 4

## References

[CR1] Abdelmohsen K, Panda AC, De S, Grammatikakis I, Kim J, Ding J, Noh JH, Kim KM, Mattison JA, de Cabo R, Gorospe M (2015). Circular RNAs in monkey muscle: age-dependent changes. Aging (albany NY).

[CR2] Bai L, Liang R, Yang Y, Hou X, Wang Z, Zhu S, Wang C, Tang Z, Li K (2015). MicroRNA-21 regulates PI3K/Akt/mTOR signaling by targeting TGFbetaI during skeletal muscle development in pigs. PLoS ONE.

[CR3] Bassel-Duby R, Olson EN (2006). Signaling pathways in skeletal muscle remodeling. Annu Rev Biochem.

[CR4] Bernhart SH, Tafer H, Muckstein U, Flamm C, Stadler PF, Hofacker IL (2006). Partition function and base pairing probabilities of RNA heterodimers. Algorithms Mol Biol.

[CR5] Chalkiadaki A, Igarashi M, Nasamu AS, Knezevic J, Guarente L (2014). Muscle-specific SIRT1 gain-of-function increases slow-twitch fibers and ameliorates pathophysiology in a mouse model of duchenne muscular dystrophy. PLoS Genet.

[CR6] Chen X, Guo Y, Jia G, Zhao H, Liu G, Huang Z (2019). Ferulic acid regulates muscle fiber type formation through the Sirt1/AMPK signaling pathway. Food Funct.

[CR7] Choi YM, Ryu YC, Kim BC (2007). Influence of myosin heavy- and light chain isoforms on early postmortem glycolytic rate and pork quality. Meat Sci.

[CR8] Fu Y, Li S, Tong H, Li S, Yan Y (2019). WDR13 promotes the differentiation of bovine skeletal muscle-derived satellite cells by affecting PI3K/AKT signaling. Cell Biol Int.

[CR9] Holloszy JO, Coyle EF (1984). Adaptations of skeletal muscle to endurance exercise and their metabolic consequences. J Appl Physiol Respir Environ Exerc Physiol.

[CR10] Hsu MT, Coca-Prados M (1979). Electron microscopic evidence for the circular form of RNA in the cytoplasm of eukaryotic cells. Nature.

[CR11] Huang M, Shen Y, Mao H, Chen L, Chen J, Guo X, Xu N (2018). Circular RNA expression profiles in the porcine liver of two distinct phenotype pig breeds. Asian-Australas J Anim Sci.

[CR12] Jeck WR, Sorrentino JA, Wang K, Slevin MK, Burd CE, Liu J, Marzluff WF, Sharpless NE (2013). Circular RNAs are abundant, conserved, and associated with ALU repeats. RNA.

[CR13] John B, Enright AJ, Aravin A, Tuschl T, Marks DS (2004). Human MicroRNA targets. PLoS Biol.

[CR14] Joo ST, Kim GD, Hwang YH, Ryu YC (2013). Control of fresh meat quality through manipulation of muscle fiber characteristics. Meat Sci.

[CR15] Kauffman RG, van Laack RL, Russell RL, Pospiech E, Cornelius CA, Suckow CE, Greaser ML (1998). Can pale, soft, exudative pork be prevented by postmortem sodium bicarbonate injection?. J Anim Sci.

[CR16] Kilkenny C, Browne W, Cuthill IC, Emerson M, Altman DG, Group NCRRGW (2010). Animal research: reporting in vivo experiments: the ARRIVE guidelines. J Gene Med.

[CR17] Kim D, Salzberg SL (2011). TopHat-Fusion: an algorithm for discovery of novel fusion transcripts. Genome Biol.

[CR18] Kim G-D, Jeong J-Y, Hur S-J, Yang H-S, Jeon J-T, Joo S-T (2010). The relationship between meat color (CIE L* and a*), myoglobin content, and their influence on muscle fiber characteristics and pork quality. Korean J Food Sci Anim Resour.

[CR19] Kim D, Pertea G, Trapnell C, Pimentel H, Kelley R, Salzberg SL (2013). TopHat2: accurate alignment of transcriptomes in the presence of insertions, deletions and gene fusions. Genome Biol.

[CR20] Kim GD, Jeong JY, Jung EY, Yang HS, Lim HT, Joo ST (2013). The influence of fiber size distribution of type IIB on carcass traits and meat quality in pigs. Meat Sci.

[CR21] Kitamura T, Kitamura YI, Funahashi Y, Shawber CJ, Castrillon DH, Kollipara R, DePinho RA, Kitajewski J, Accili D (2007). A Foxo/Notch pathway controls myogenic differentiation and fiber type specification. J Clin Invest.

[CR22] Kristensen LS, Andersen MS, Stagsted LVW, Ebbesen KK, Hansen TB, Kjems J (2019). The biogenesis, biology and characterization of circular RNAs. Nat Rev Genet.

[CR23] Larzul C, Lefaucheur L, Ecolan P, Gogue J, Talmant A, Sellier P, Le Roy P, Monin G (1997). Phenotypic and genetic parameters for longissimus muscle fiber characteristics in relation to growth, carcass, and meat quality traits in large white pigs. J Anim Sci.

[CR24] Lefaucheur L (2010). A second look into fibre typing–relation to meat quality. Meat Sci.

[CR25] Legnini I, Di Timoteo G, Rossi F, Morlando M, Briganti F, Sthandier O, Fatica A, Santini T, Andronache A, Wade M, Laneve P, Rajewsky N, Bozzoni I (2017). Circ-ZNF609 is a circular RNA that can be translated and functions in myogenesis. Mol Cell.

[CR26] Liang D, Wilusz JE (2014). Short intronic repeat sequences facilitate circular RNA production. Genes Dev.

[CR27] Liang G, Yang Y, Niu G, Tang Z, Li K (2017). Genome-wide profiling of Sus scrofa circular RNAs across nine organs and three developmental stages. DNA Res.

[CR28] Liu L, Yao L, Wang S, Chen Z, Han T, Ma P, Jiang L, Yuan C, Li J, Ke D, Li C, Yamahara J, Li Y, Wang J (2019). 6-gingerol improves ectopic lipid accumulation, mitochondrial dysfunction, and insulin resistance in skeletal muscle of ageing rats: dual stimulation of the AMPK/PGC-1alpha signaling pathway via plasma adiponectin and muscular AdipoR1. Mol Nutr Food Res.

[CR29] Ogata H, Goto S, Sato K, Fujibuchi W, Bono H, Kanehisa M (1999). KEGG: kyoto encyclopedia of genes and genomes. Nucleic Acids Res.

[CR30] Renerre M (1990). Review: factors involved in the discoloration of beef meat. Int J Food Sci Technol.

[CR31] Ryu YC, Kim BC (2006). Comparison of histochemical characteristics in various pork groups categorized by postmortem metabolic rate and pork quality. J Anim Sci.

[CR32] Schachter TN, Shen T, Liu Y, Schneider MF (2012). Kinetics of nuclear-cytoplasmic translocation of Foxo1 and Foxo3A in adult skeletal muscle fibers. Am J Physiol Cell Physiol.

[CR33] Shen L, Gan M, Tang Q, Tang G, Jiang Y, Li M, Chen L, Bai L, Shuai S, Wang J, Li X, Liao K, Zhang S, Zhu L (2019). Comprehensive analysis of lncRNAs and circRNAs reveals the metabolic specialization in oxidative and glycolytic skeletal muscles. Int J Mol Sci.

[CR34] Stitt TN, Drujan D, Clarke BA, Panaro F, Timofeyva Y, Kline WO, Gonzalez M, Yancopoulos GD, Glass DJ (2004). The IGF-1/PI3K/Akt pathway prevents expression of muscle atrophy-induced ubiquitin ligases by inhibiting FOXO transcription factors. Mol Cell.

[CR35] Sun J, Xie M, Huang Z, Li H, Chen T, Sun R, Wang J, Xi Q, Wu T, Zhang Y (2017). Integrated analysis of non-coding RNA and mRNA expression profiles of 2 pig breeds differing in muscle traits. J Anim Sci.

[CR36] Veno MT, Hansen TB, Veno ST, Clausen BH, Grebing M, Finsen B, Holm IE, Kjems J (2015). Spatio-temporal regulation of circular RNA expression during porcine embryonic brain development. Genome Biol.

[CR37] Wang L, Feng Z, Wang X, Wang X, Zhang X (2010). DEGseq: an R package for identifying differentially expressed genes from RNA-seq data. Bioinformatics.

[CR38] Wang H, Feng C, Jin Y, Tan W, Wei F (2019). Identification and characterization of circular RNAs involved in mechanical force-induced periodontal ligament stem cells. J Cell Physiol.

[CR39] Wang J, Ge J, Cao H, Zhang X, Guo Y, Li X, Xia B, Yang G, Shi X (2019). Leptin promotes white adipocyte browning by inhibiting the Hh signaling pathway. Cells.

[CR40] Wang T, Xu YQ, Yuan YX, Xu PW, Zhang C, Li F, Wang LN, Yin C, Zhang L, Cai XC, Zhu CJ, Xu JR, Liang BQ, Schaul S, Xie PP, Yue D, Liao ZR, Yu LL, Luo L, Zhou G, Yang JP, He ZH, Du M, Zhou YP, Deng BC, Wang SB, Gao P, Zhu XT, Xi QY, Zhang YL, Shu G, Jiang QY (2019). Succinate induces skeletal muscle fiber remodeling via SUNCR1 signaling pathway. EMBO Rep.

[CR41] Wang K, Gao XQ, Wang T, Zhou LY (2021). The function and therapeutic potential of circular RNA in cardiovascular diseases. Cardiovasc Drugs Ther.

[CR42] Wei X, Li H, Yang J, Hao D, Dong D, Huang Y, Lan X, Plath M, Lei C, Lin F, Bai Y, Chen H (2017). Circular RNA profiling reveals an abundant circLMO7 that regulates myoblasts differentiation and survival by sponging miR-378a-3p. Cell Death Dis.

[CR43] Yu M, Wang H, Xu Y, Yu D, Li D, Liu X, Du W (2015). Insulin-like growth factor-1 (IGF-1) promotes myoblast proliferation and skeletal muscle growth of embryonic chickens via the PI3K/Akt signalling pathway. Cell Biol Int.

[CR44] Zhang XO, Wang HB, Zhang Y, Lu X, Chen LL, Yang L (2014). Complementary sequence-mediated exon circularization. Cell.

[CR45] Zhong Z, Huang M, Lv M, He Y, Duan C, Zhang L, Chen J (2017). Circular RNA MYLK as a competing endogenous RNA promotes bladder cancer progression through modulating VEGFA/VEGFR2 signaling pathway. Cancer Lett.

